# Survey data on ventilation provision and use in homes in Great Britain

**DOI:** 10.1016/j.dib.2025.112090

**Published:** 2025-09-19

**Authors:** Cairan A. Van Rooyen, Tim Sharpe

**Affiliations:** a The Bartlett School of Environment, Energy and Resources, University College London, Central House, 14 Upper Woburn Place, London WC1H 0NN, United Kingdom; b Department of Architecture, University of Strathclyde, Level 3, James Weir Building, 75 Montrose Street, Glasgow G1 1XJ United Kingdom

**Keywords:** Housing, Mechanical ventilation, Damp, Trickle vents, Mould, Indoor environmental quality, Building standards

## Abstract

This article describes data collected from an online questionnaire survey of 2, 039 adults in England, Wales, and Scotland (Great Britain) to assess the provision and use of mechanical ventilation, trickle vents, and windows in their homes. The survey was deployed on the 17th of June 2022 and collected data across four categories: socioeconomic and demographic characteristics, dwelling features, ventilation practices, and other contextual factors. The questionnaire included 65 questions and had a median completion time of 12 minutes, with all respondents completing the survey.

The dataset is broadly representative of the British population and housing typologies. It provides valuable insights into the relationships between dwellings, occupants, their ventilation provision, and behaviours. The data is stored in a comma-separated values (.csv) file containing 396 variables, with responses formatted as binary, continuous, discrete, categorical, and free-text.

This dataset can be utilised by academics for indoor environmental quality and energy modelling to refine assumptions about ventilation provision and practices. Public health professionals can use the data to estimate the health impacts of exposure to indoor pollutants, which can result from poor ventilation provision and to develop targeted health information. Government can use this evidence to inform policies and strategies aimed at improving ventilation in existing homes. The dataset is accessible through the Mendeley Data repository.

Specifications Table

**Table:** 

Subject	Engineering & Materials Science
Specific subject area	Ventilation provision and use in homes in Great Britain - including relevant contextual data (socioeconomic, demographic, physical dwelling characteristics, and services).
Type of data	Table (dataset.csv) contains raw data from questionnaire survey from 2, 039 adults in England, Wales, and Scotland. Table (variable_file.xlsx) contains a description of all variables, valid ranges, missing data codes, and frequency distribution of variables in the dataset.csv. Document (questionnaire.docx) contains the complete questionnaire that was used to collect the data in dataset.csv.
Data collection	Data was collected using an established survey organisation (YouGov). An online questionnaire was issued to a sub-sample of their panel of 2.6 million adults on the 17th of June 2022. The resulting sample contains responses from 2, 039 adults, with a median time to complete the survey of 12 min. The survey design and deployment method resulted in complete responses from all participants. The sample is broadly representative of the Great British population and housing stock.
Data source location	Data was collected from 2, 039 adults in England, Wales, and Scotland (Great Britain) using a panel from YouGov.
Data accessibility	Repository name: Mendeley data Data identification number: 10.17632/x3d5xnsypd.2 Direct URL to data: https://data.mendeley.com/datasets/x3d5xnsypd/2
Related research article	[1] Van Rooyen, C. and Sharpe, T., 2024. Ventilation provision and use in homes in Great Britain: A national survey. Building and Environment, 257, p.111528. https://doi.org/10.1016/j.buildenv.2024.111528

## 1 Value of the Data

• This represents the first publicly available, large-scale dataset documenting ventilation provision, behaviours, and contextual factors in British homes, containing 396 variables across sociodemographic, dwelling, behavioural, and contextual categories.
• Researchers can use this data to inform indoor environmental quality and energy modelling and to refine assumptions about ventilation provision and practices and explore the relationships between socioeconomic factors, ventilation provision, and ventilation behaviours.
• The data can enable public health professionals to estimate the health impacts of poor ventilation, identify at-risk populations, and develop targeted health messaging.
• Policymakers and regulators can use the data to develop and shape policies and regulations aimed at improving ventilation provision and standards in existing homes. Additionally, the data can be used as evidence to support strategies to improve compliance with building regulations in new homes.
• The dataset includes comprehensive and detailed information on socioeconomic and demographic characteristics, dwelling features, ventilation practices, and other contextual factors.
• Although not strictly representative of the British population or housing stock, the large sample size and detailed data provide valuable insights that are highly relevant for understanding ventilation behaviours and their implications.

## 2 Background

People in Britain spend 95 % of their time indoors and most of this time in their homes [2]. Whilst indoors, occupants are exposed to the indoor environment, which can impact comfort, health, and well-being [3]. Ventilation can modify occupants’ exposure to indoor pollutants and moisture; although ventilation can also lead to heat loss, increased energy use, and carbon emissions [[4], [5], [6]]. The need to upgrade British homes to abate climate change is evident [7]. However, energy efficiency upgrades can lead to increases in airtightness, reducing adventitious ventilation, and if ventilation provision is absent, this may result in an increased risk of creating harmful conditions inside homes [[8], [9], [10], [11]]. Research studies of ventilation provision and use in Britain and the UK are dated and typically use a case study approach [12,13]. The result is that current ventilation provision and use were poorly understood and characterised in homes across Great Britain. Given the importance of ventilation for health, energy, and the climate, there is a need to close this knowledge gap. This data article adds value to the published article by including much more data and variables that were not presented in the published article. This additional data can enable further research using this dataset.

## 3 Data Description

The dataset comprises three files: the questionnaire (questionnaire.docx), the variable file (variable_file.xlsx), and the dataset (dataset.csv). Each file is structured to provide comprehensive information on the survey and its results.

1. Questionnaire (questionnaire.docx)

This file contains the full questionnaire issued to respondents. Note the original questionnaire was issued to respondents using YouGov’s online platform. The file includes:

- Base Questions: core questions asked of all respondents.
- Routing Information: details on how respondents were directed to different questions based on their previous answers.
- Question Types: information on whether questions were multiple-choice, open-text, or required specific formats (e.g., numerical input).

2. Variable File (variable_file.xlsx)

This file provides details of each of the 396 variables in the dataset. It includes six columns:

i. Variable Name: the name of each variable as it appears in the dataset.
ii. Variable Type: the type of data (e.g., binary, continuous, discrete, categorical, free text).
iii. Variable Description: a brief description of what each variable represents.
iv. Valid Ranges: the ranges of data for each variable. For categorical variables, this lists the categories.
v. Missing Data Codes: the number of missing data, grouped by their type, for each variable.
vi. Frequency Distribution: unique value counts for each variable.

3. Dataset (dataset.csv)

This comma-separated values file contains the raw data collected from the survey. It includes:

- Header Row: the first row contains the variable names.
- Data Rows: each subsequent row represents the responses from an individual participant. The dataset includes 2, 039 rows (one for each respondent) and 396 columns (one for each variable).

## 4 Experimental Design, Materials and Methods

1. Contribution and purpose

This data article provides detailed documentation and describes access to the complete dataset from the research paper published in Building and Environment [1]. While the original paper focused on specific research questions regarding ventilation provision and compliance with building standards, this data article serves three purposes:

1. Dataset documentation: The original research analysed a subset of the 396 variables collected. This data article provides documentation of all variables, including detailed descriptions, coding schemes, and data structures that enable secondary analysis.
2. Data accessibility: This article provides structured access to the raw data through the Mendeley Data repository, complete with variable files and questionnaire documentation to facilitate independent research.
3. Further research potential: The dataset supports exploring research questions beyond those addressed in the original paper.

2. Questionnaire design

To characterize ventilation provision and use in homes in Great Britain a detailed questionnaire was developed based on a review of the literature.

This questionnaire survey collected four categories of data:

1.1 Socioeconomic and demographic information on the household was collected in previous research and is a key aspect of characterising the household and its occupants. The questionnaire used similar or adapted questions from other research to collect information on the household and its occupants [12,[14], [15], [16]]. Examples of data collected in this category were: age, gender, ethnicity, employment status, marital status, number of people and children in household, parental status, use of social media, number of smokers, tenure type, duration living in home, number of people in each bedroom.
1.2 Information was collected on the physical dwelling and its services, with a focus on ventilation provisions. This information was key to answering the research questions and addressing the gap in literature. Examples of data collected in this category were: dwelling type, construction date, heating systems, number of bedrooms, postcode, trickle vents, mechanical ventilation and controls, presence of IAQ sensor.
1.3 The literature describes occupants’ behaviour to be one of the most important factors influencing ventilation performance in homes, therefore, information was collected from each respondent on their use of ventilation, including barriers and drivers for its use [12,17,18]. Examples of data collected in this category were: trickle vent use and operation in all rooms, window opening behaviours, impact of COVID on ventilation behaviours, impact of IAQ sensor on ventilation behaviours and awareness of IAQ, drivers and barriers for trickle vents, mechanical ventilation and windows.
1.4 Other contextual and exploratory questions were included, based on their expected relationship with ventilation behaviour [19]. Examples of data collected in this category were: health conditions affecting ventilation, type and source of ventilation advice received, perceptions of indoor air quality, pollution and thermal comfort, presence of mould and damp, and bedroom door use.

3. Deployment

This survey was deployed using YouGov, which is an established survey organisation with a panel of 2.6 million adults. YouGov initially drew a representative (gender, age, employment status, and social grade) sub-sample and invited that sub-sample to respond to the questionnaire. Before the respondents took part, they were screened again to ensure representativeness of the sample. If they met the criteria they were informed about the nature of the survey, duration to complete, and reward. At this point each participant was free to opt in or opt out of the survey. As panel members, YouGov holds information on the participants including socio demographic and some household data. This information reduced survey time and associated survey fatigue.

The survey was deployed on the 17th of June 2022 using YouGov’s online platform. The resulting sample is 2, 039 adults from England, Wales, and Scotland. All respondents completed all questions and the median time to complete was 12 min.

The resulting sample was compared with population data for dwelling age, dwelling type, tenure, heating type, respondent age, respondent gender, respondent employment status, and household social grade – this analysis is presented in Appendix B of the Ventilation provision and use in homes in Great Britain paper [1]. The sample is not strictly representative of the Great British housing stock, but given the large sample size, it is highly relevant.

4. Software environment

Python Version: 3.12.7
pandas 2.2.2
numpy 1.26.4
scipy 1.13.1 - chi-square tests
statistics

5. Data transformations and cleaning procedures

Initial data processing involved converting all data to suitable data types, with categorical and yes/no variables converted to binary using mapping functions. Several derived variables were created to facilitate analysis: building age classifications included Boolean variables identifying homes built before 1980 (pre_1980) and 1990 (pre_1990) using string pattern matching. Mechanical ventilation variables comprised composite indicators for kitchen (kitchen_mech_vent_any) and bathroom (bathroom_mech_vent_any) mechanical ventilation, plus a combined variable (mech_kit_bath) indicating homes with both kitchen and bathroom mechanical ventilation or whole-house systems (MVHR/PIV). Ventilation provision analysis used Boolean variables for trickle vent status across all rooms (trickle_vent_open_all_rooms) and identification of any closed or absent trickle vents (tv_closed_anyroom). Window opening behaviour was captured through four Boolean variables (windows_opened_regularly_[day/night]_[summer/winter]) representing regular opening patterns defined as `All the time', `Daily', or `A few times a week'. Information and composite variables included Boolean indicators for receipt of any ventilation information (ventilation_information_any), combined mechanical ventilation with trickle vents (mech_kit_bath_tv), and a triple combination including information receipt (mech_kit_bath_tv_info).

6. Missing data handling

There is no missing data and there are complete responses from all participants. To some questions, participants had the option to respond “Don't know” and/or “Prefer not to say”. Furthermore, there are also a range of open text responses to certain questions.

7. Data quality assessment and exclusion criteria

Data exclusions were applied to ensure validity: responses with completion times of 5 min or less were excluded as this indicated insufficient time for thoughtful responses. Dwelling types including `Static Caravan/mobile home/trailer', `Don't know', `Other', and `Prefer not to say' were excluded as these building types were inappropriate for ventilation analysis in respect of building regulations. Additionally, mutually exclusive responses were removed where cases reported both `intermittent' and `continuous' ventilation in the same space.

## Limitations

The accuracy of the results from the questionnaire are dependent on the respondents' self-reported ventilation provision and use. This introduces potential sources of error and biases related to the respondents' knowledge and awareness of their ventilation systems and practices. To mitigate this, the questionnaire was designed using plain language, supplemented with figures and descriptive prompts, and allowed respondents to indicate if they did not know the answer rather than guessing.

The data collected did not include any inspection or validation of the reported ventilation systems and practices. Future studies could incorporate physical surveys or monitoring to verify the self-reported data.

The survey was conducted once during the summer, and this timing may also introduce biases. Respondents were asked to recall their ventilation practices during different seasons, which could lead to recall bias.

The survey is based on a broadly representative sample which, although large, is not strictly representative of the British population or housing stock. The dataset has subtle differences related to the demographic and socioeconomic characteristics of the sample. However, the sample was compared to categorisations of housing and tenure and was broadly representative.

Lastly, the YouGov system and consequently the survey design and deployment through an online platform may exclude certain populations, such as those without internet access or those less comfortable with digital technologies. This could result in under-representation of certain groups.

## Ethics Statement

The survey was administered by YouGov, an established survey organisation, which managed the recruitment and data collection processes. All participants were members of the YouGov panel, which consists of individuals who have agreed to participate in surveys and have provided informed consent for their data to be used for research purposes.

Before participating in the survey, respondents were informed about the nature of the study, the estimated time required to complete the questionnaire, and the incentives for participation. Participation was entirely voluntary, and respondents had the option to opt out at any stage without any consequences.

YouGov ensured that the survey adhered to ethical standards and data protection regulations, including the General Data Protection Regulation (GDPR). All data collected was anonymised to protect the privacy of the respondents. Personal identifiers were removed, and the data was stored securely to prevent unauthorized access.

The study did not involve the collection of any sensitive personal data, and the questions were designed to minimize any potential discomfort or distress to the participants. YouGov rely on ‘legitimate interests’ as the legal grounds for processing this data.

## CRediT authorship contribution statement

**Cairan A. Van Rooyen:** Conceptualization, Methodology, Software, Validation, Formal analysis, Investigation, Resources, Data curation, Writing – original draft, Writing – review & editing, Project administration. **Tim Sharpe:** Conceptualization, Methodology, Formal analysis, Investigation, Resources, Writing – review & editing, Supervision, Project administration, Funding acquisition.

## Data Availability

Mendeley DataDataset from the survey of ventilation provision and use in homes in Great Britain (Original data).
